# Real-world inter-observer variability of the sequential organ failure assessment (SOFA) score in intensive care medicine: the time has come for an update: authors’ reply

**DOI:** 10.1186/s13054-023-04468-9

**Published:** 2023-05-11

**Authors:** Rui Moreno, Andrew Rhodes, Mervyn Singer, Jean-Louis Vincent, On behalf of the authors.

**Affiliations:** 1 Hospital de São José, Centro Hospitalar Universitário de Lisboa Central, Faculdade de Ciências Médicas de Lisboa, Nova Medical School, Lisbon, Portugal; 2 Faculdade de Ciências da Saúde, Universidade da Beira Interior, Covilhã, Portugal; 3 Adult Critical Care, St. George’s University Hospitals NHS Foundation Trust, St. George’s University of London, London, UK; 4 Division of Medicine, Bloomsbury Institute of Intensive Care Medicine, University College London, London, UK; 5 Department of Intensive Care, Erasme Hospital, Université Libre de Bruxelles, 1070 Brussels, Belgium

Dear Editor,

Thank you for the opportunity to reply to the letter from Pérez-Torres et al. regarding our manuscript [1]. Pérez-Torres supports our main contention that it is time to update the SOFA score to ensure it fulfils current requirements following the many changes in clinical practice over the last three decades.

The example provided is self-explanatory: in 1996, the clinical use of veno-venous extracorporeal membrane oxygenation (VV-ECMO) was virtually non-existent. Consequently, it was not considered within the respiratory dysfunction score. If the original rules described in the 1996 manuscript are applied, receiving VV-ECMO would score 3 points. If the rules are modified to incorporate VV-ECMO as a marker of greatest dysfunction, then the score would be 4. The goalposts have shifted.

A useful analogy is to compare a mathematical model to a molecule used for therapy. A change, even minor, in a single atom may result in no effect yet the molecule is different. Only after successful testing can it be implemented into clinical practice. This principle is not widely applied to mathematical models, even if the scientific reasoning is the same. We agree that it is time to change, and that the accumulation of these “small changes” was why we proposed the development and validation of a new “SOFA 2.0” score instead of a modification of the original system with the same name [3].

Thank you for raising the attention to this important yet grossly underestimated problem.

## Data Availability

Not Applicable.
